# The Safety and Efficacy of Cryotherapy in the Prevention of Paclitaxel-Induced Neuropathy: A Systematic Review

**DOI:** 10.7759/cureus.44026

**Published:** 2023-08-24

**Authors:** Md Fahad Hossain, Manish Kharel, Mostafa Hasan Rajib, Mahfuza A Khan, Md. Yasin Anwar, Yogesh Lamsal, Syed Nurul Aziz

**Affiliations:** 1 Hospital Medicine, Ministry of Health, Upazila Health Complex, Kishoreganj, BGD; 2 Medicine and Surgery, Jahurul Islam Medical College, Bhagalpur, BGD; 3 Endocrinology, Bangabandhu Sheikh Mujib Medical University (BSMMU), Dhaka, BGD; 4 Internal Medicine, Sylhet M.A.G (Muhammad Ataul Goni) Osmani Medical College, Sylhet, BGD; 5 Cardiology, 250 Bed District Sadar Hospital, Cox Bazar, BGD; 6 Emergency Medicine, Sahara Hospital Pvt. Ltd., Pokhara, NPL; 7 Obstetrics and Gynaecology, University of Missouri, Columbia, USA; 8 Internal Medicine, Shaheed Suhrawardy Medical College, Dhaka, BGD

**Keywords:** non-pharmacological interventions, cancer patients, paclitaxel, cryotherapy, peripheral neuropathy

## Abstract

The chemotherapeutic agent paclitaxel has significantly enhanced the treatment of various types of cancer. However, the quality of life of cancer patients is often impacted by the painful and dose-restrictive paclitaxel side effect known as paclitaxel-induced peripheral neuropathy (PIPN). A non-pharmacological method called cryotherapy has shown promise in alleviating PIPN-related symptoms. In this systematic review, we aimed to evaluate the safety and effectiveness of cryotherapy in preventing PIPN. The review analyzed four randomized controlled trials (RCTs) involving individuals treated with paclitaxel for breast and gynecological cancer. Cryotherapy showed success in lowering PIPN symptoms in several studies, as judged by various outcome measures, although the findings varied. The safety profile of cryotherapy was typically good, with minimal side effects. However, methodological variations and small sample sizes in the studies analyzed limit drawing definitive conclusions from them. To obtain conclusive evidence, studies with standardized techniques and larger sample sizes are required. Further research is necessary to understand cryotherapy's potential mechanisms and long-term effects. This review highlights the potential of cryotherapy in the management of PIPN, explains how it works, and suggests future research topics to improve its application.

## Introduction and background

The powerful chemotherapeutic drug called paclitaxel has totally transformed how cancer is treated and has had a positive impact on many different types of cancer [1]. However, paclitaxel-induced peripheral neuropathy (PIPN), an unpleasant and dose-limiting side effect, frequently compromises its efficacy [1]. Cancer patients' quality of life is significantly impacted by this illness, which causes pain, numbness, tingling, and loss of sensation in the extremities [1,2]. The prevalence of PIPN can vary, depending on the type of cancer being treated, the precise dose regimens employed, and the unique patient features. Numerous studies have shown various PIPN prevalence rates. For instance, it has been reported that 60-70% of breast cancer patients have PIPN following paclitaxel-based treatment [3][4]. The frequency of PIPN varies from 30 to 85% in ovarian cancer patients receiving paclitaxel treatment [5,6]. The frequency of PIPN has been estimated to range between 30 and 40% in lung cancer patients receiving paclitaxel-based therapy [7,8]. Finding efficient strategies to manage PIPN has emerged as a crucial focus of research as more patients receive paclitaxel-based therapy [2].

Cryotherapy has emerged as a viable non-pharmacological treatment among these therapies, garnering interest for its potential efficacy and safety in reducing PIPN-related symptoms [9]. Cold temperatures are carefully applied to a particular location of the body during cryotherapy, sometimes referred to as cold therapy [9]. This therapeutic approach has been used in medicine for various purposes, including pain control and inflammation reduction [9]. While cryotherapy may be effective in treating PIPN, its underlying mechanisms are still not fully understood [10]. On the other hand, it is hypothesized that the use of cool temperatures could lessen neural inflammation, stifle axon deterioration, and improve nerve conduction velocity [10]. The neuroprotective benefits of cryotherapy may also be influenced by changes in cytokine release and oxidative stress [10].

In clinical practice, ensuring the safety of any therapeutic intervention is crucial [11]. When performed properly, cryotherapy is generally regarded as safe with few negative effects [9,11]. However, in some patient populations, such as those with Raynaud's phenomenon or cold hypersensitivity, utmost caution is warranted in terms of employing cryotherapy, or they may require other methods [11,12]. It is imperative to evaluate the safety profile of cryotherapy specifically in the setting of PIPN, particularly in light of the risk of neuropathic consequences [12]. Cryotherapy has demonstrated encouraging results in preclinical research investigating its effectiveness in treating animal models of neuropathy [13]. Researchers have observed changes in nerve shape, decreased pain responses, and increased nerve regeneration following cryotherapy [13]. However, further research is needed to validate these findings and apply them in clinical contexts [13].

The effect of cryotherapy on PIPN has been the subject of extensive clinical research [14]. Cryotherapy combined with paclitaxel treatment has been shown to significantly reduce neuropathic pain and enhance sensory function in some patients [14,15]. However, it is difficult to establish firm conclusions due to variations in study designs, patient demographics, and outcome measures [15]. While cryotherapy for the treatment of PIPN has some potential, there are still some challenges associated with it [16]. Focused research is required to determine the best cryotherapy methods, comprehend how each patient responds differently to cryotherapy, and examine its long-term implications [16].

## Review

Methodology

In performing this systematic review, we adhered to the Preferred Reporting Items for Systematic Reviews and Meta-Analyses (PRISMA) guidelines [17]. 

Data Sources

We conducted searches on a number of databases, including MEDLINE through Pubmed, Web of Science, the Cochrane Library (Cochrane Central Register of Controlled Trials-CENTRAL), EBSCOhost, and Scopus with a search strategy that involved using the keywords ‘cryotherapy’, ‘Cryotherapies’, ‘Cold Therapy’, ‘Cold Therapies’, ‘low-temperature procedure’, ‘cryotherapy device’, ‘ice’, ‘hypothermia procedures’, ‘Paclitaxel’, ‘Anzatax,’ ‘NSC-125973’, ‘Taxol’, ‘Paxene’, ‘Onxol’, ‘Bris’, ‘Peripheral Nervous System Diseases’, ‘Peripheral Nervous System Disorders’, ‘Peripheral Neuropathy’, ‘Peripheral Neuropathies’. The search was conducted on July 10, 2023.

Study Selection

We looked for publications that examined the advantages and drawbacks of cryotherapy for preventing peripheral neuropathy caused by paclitaxel. We only included randomized controlled trials (RCTs) published in the English language. Studies that did not take cryotherapy into account, studies not focused on PIPN, lab experiments, unfinished or continuing investigations, animal studies, reviews, case series, case reports, letters, comments, editorials, book chapters, and opinion pieces were excluded.

Two reviewers each examined the title and abstract of the papers that were elicited from the search, and any discrepancies were settled by a third reviewer. We utilized the online resource Rayyan to filter the articles [18]. The full-text papers were then examined by two separate teams, with any disagreements being resolved by the lead reviewer. We excluded the articles by using the "prioritization and sequential exclusion" approach [19]. The reasons for the exclusion were documented.

Data Extraction

The study population, the length and dose of the intervention, the positive and negative outcomes, the seriousness of the negative results, and other aspects were taken into account when compiling the data. Data were separately retrieved by two reviewers, and any disagreements were settled by cross-checking by the main reviewer.

Data Analysis

We opted for a narrative synthesis of the data. A meta-analysis was not possible because of the wide range of the results obtained.

Risk of Bias Assessment

The risk of bias (ROB) was assessed using the Cochrane Risk of Bias assessment technique. Two sets of reviewers separately assessed the ROB, and any discrepancies were resolved through discussion. The domains taken into account in terms of ROB included random sequence generation, participant and personnel blinding, allocation concealment, blinding of outcome assessors, insufficient outcome data, selective reporting, and other biases.

Results

Search Results

**Figure 1:**
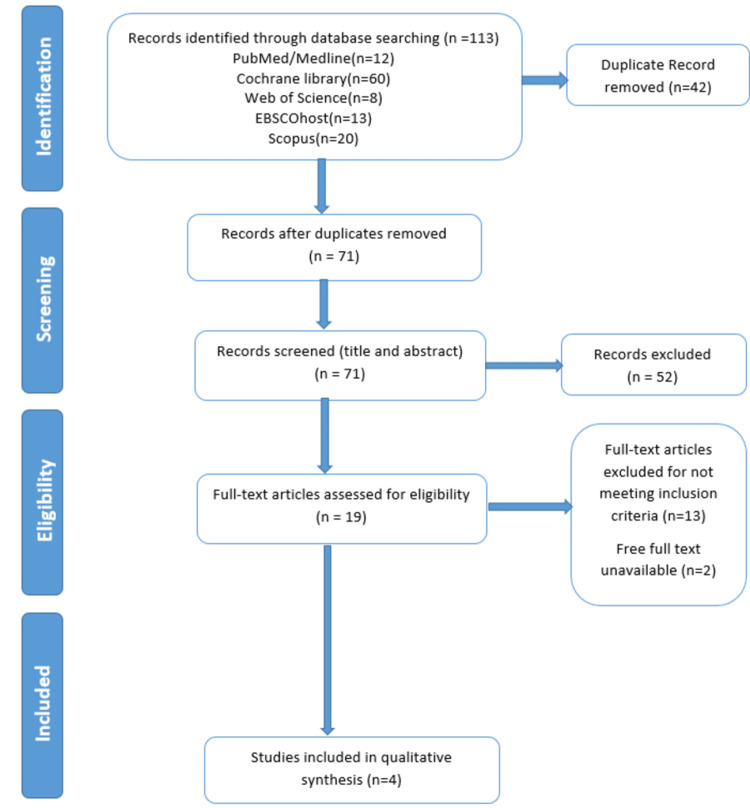
PRISMA flow diagram depicting the study selection process PRISMA: Preferred Reporting Items for Systematic Reviews and Meta-Analyses

A thorough search across five databases elicited a total of 113 studies. We listed 71 articles for title and abstract screening after eliminating 42 duplicates; 52 items were further excluded at this point for failing to meet the standards outlined in the methodology section for inclusion. Fifteen of the remaining papers were disqualified at the full-text screening stage, as they were not pertinent to the research intervention, leaving us with four studies for the final analysis. Figure 1 depicts the detailed PRISMA flow diagram of the article selection procedure.

ROB Assessments of Clinical Trials

**Figure 2:**
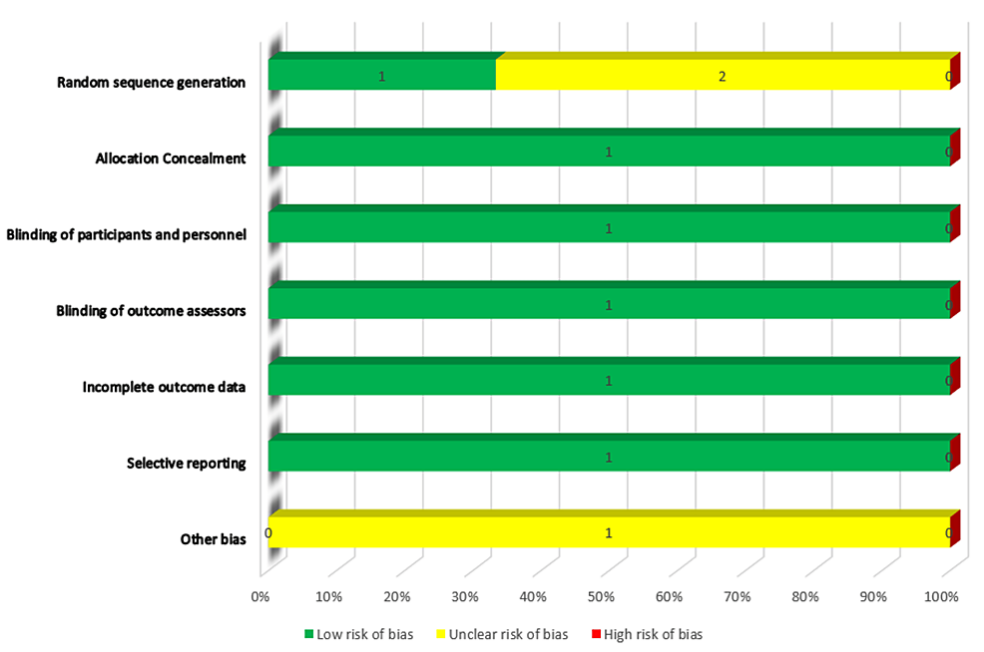
Graphical presentation of ROB (risk of bias) assessment of included studies

We used the ROB tool to assess the risk of bias. All articles provided information about random sequence generation. Allocation concealment was done for all the articles. All the participants and personnel were also blinded about the outcome accessor of data, and hence we rated them as low risk of bias. No articles had incomplete outcome data or there was no selective reporting done, and the risk of bias was low in this regard as well. As there was no information about any other bias risk, we classified all articles as unclear risk for other biases. Figure 2 shows the graphical presentation of ROB.

Characteristics of Included Studies

All the studies included in this systematic review were RCTs where paclitaxel was given for a variety of cancers and cryotherapy was used for the prevention of paclitaxel-induced neuropathy. All studies were conducted between 2019 and 2022 and involved a total of 215 patients with a mean age >50 years. The country of origin of the studies was as follows: the USA (Ruddy et al., 2019 [20]), Japan (Shigematsu et al., 2019 [21]), Singapore (Ng et al., 2020 [22]), and Thailand (Chitkumarn et al., 2022 [23]). All the trials included both genders. Cryotherapy was usually compared with the standard therapy that was used to prevent neuropathy. The intervention lasted for 12 weeks in all studies. Paclitaxel-induced neuropathy was analyzed mainly by employing patient-reported questionnaires/tools like the Functional Assessment of Cancer Therapy-Neurotoxicity (FACT-NTX) score, the Patient Neurotoxicity Questionnaire (PNQ), and the European Organization for Research and Treatment of Cancer Quality of Life Questionnaire Chemotherapy-Induced Peripheral Neuropathy Scale 20 (EORTC QLQ-CIPN20).

Description of Intervention

The cryotherapy method predominantly used in the studies comprised frozen gloves or stockings, except for the study by Ruddy et al. where they utilized crushed ice. The cryotherapy usually started 15 minutes prior to the initiation of each paclitaxel dose and finished 15 minutes following the completion of each dose, mostly for a total duration of 90 minutes for 12 weeks.

Description of Outcomes

Table 1 demonstrates the detailed outcomes of the intervention with dosage-related data and summary.

**Table 1:** Characteristics of the included studies ECOG: the Eastern Cooperative Oncology Group; EORTC QLQ-CIPN20: the European Organization for Research and Treatment of Cancer Quality of Life Questionnaire Chemotherapy-Induced Peripheral Neuropathy Scale 20; FACT-NTX: Functional Assessment of Cancer Therapy-Neurotoxicity; NCI-CTCAE: the National Cancer Institute-Common Terminology Criteria for Adverse Events; FACT: Functional Assessment of Cancer Therapy; CTCAE: Common Terminology Criteria for Adverse Events; PNQ: Patient Neurotoxicity Questionnaire; PRO: Patient-reported Outcome; CIPN20: Chemotherapy-Induced Peripheral Neuropathy Scale 20; FACT/GOG-Ntx: Functional Assessment of Cancer Therapy/Gynecologic Oncology Group—Neurotoxicity

Study	Components	Features
Ruddy et al., 2019 [20]	Study design	Randomized controlled trial
	Summary	46 breast cancer patients were randomized to receive standard care or topical cryotherapy, which involved applying bags of crushed ice to the hands and feet for 12 weeks. The patients had to have a life expectancy of at least six months and an ECOG performance status of 0-1 without any prior history of peripheral neuropathy or a known cause. Patients received the EORTC QLQ-CIPN20 on paper at baseline, prior to each dosage of paclitaxel, as well as every 30 days for six months following the conclusion of the 12-week paclitaxel course
	Population	46 breast cancer patients; intervention: 23, control: 23
	Intervention	Cryotherapy (bags of crushed ice) began 15 minutes before the start of each paclitaxel treatment and ended 15 minutes after the end of each dose
	Outcome	Safety and efficacy: The difference in EORTC QLQ-CIPN20 sensory scores throughout the course of the 12 weeks of paclitaxel treatment between the two arms was not statistically significant, with a mean of 3.45 (95% confidence interval = (3.13, 10.02); p=0.26). A Wilcoxon rank-sum p-value of 0.01 indicated that there was less neuropathy in our cryotherapy arm than in the combined control arms when the cryotherapy arm of the current trial was compared to the combined control arm (minocycline and pregabalin) during the 12-week treatment period and for 6 months after treatment completion. Adverse effects: No episodes of frostbite, but there was some discomfort related to numbness/tingling and redness/irritation of the skin
	Limitation	Small sample size; ethnically homogeneous patient population (only white patients included)
Shigematsu et al., 2019 [21]	Study design	Randomized phase II controlled trial
	Summary	44 patients with breast cancer receiving 12 weekly doses of paclitaxel were randomly assigned (1:1) to either the cryotherapy or control group. According to a recent report on cryotherapy, patients underwent continuous freezing (20 °C) treatment with Elastogel, mittens, and slippers on their hands and feet from 15 minutes before to paclitaxel infusion until 15 minutes after infusion (a total of 90 minutes). The proportion of patients whose FACT-NTX score had significantly decreased served as the primary objective. PNQ, NCI-CTCAE for peripheral neuropathy, and the FACT-Taxane scores served as the secondary outcomes
	Population	44 patients; cryotherapy: 22, control group: 22
	Intervention	Fifteen minutes before the commencement of each dose of paclitaxel and 15 minutes after the end of each treatment (a total of 90 minutes) were spent doing cryotherapy using frozen gloves and socks.
	Outcome	Safety and efficacy - primary endpoint: FACT-NTX score. Those who saw a noticeable decline in FACT-NTX scores were considerably underrepresented in the cryotherapy group compared to the control group (41 vs. 73%, p=0.03). Secondary endpoint: The incidence of CTCAE grade 2 sensory and motor peripheral neuropathy, PNQ grade D or higher for sensory peripheral neuropathy, and a decline in the FACT-Taxane score were all significantly lower in the cryotherapy group than in the control group (p=0.001). Adverse effects: Cryotherapy did not have any negative side effects that were life-threatening
	Limitation	Small sample size focused on weekly-paclitaxel-based regimens only
Ng et al., 2020 [22]	Study design	A prospective, parallel assignment, randomized controlled study conducted at the National Cancer Centre, Singapore
	Summary	46 breast cancer patients were randomly assigned (1:1) to cryotherapy or control groups to receive 12 weekly doses of paclitaxel treatment. For cryotherapy, Elastogel hypothermia gloves and socks were applied to the hands and feet for 90 minutes, starting 15 minutes prior to the infusion of paclitaxel and ending 15 minutes after. Electrophysiological tests and PRO questionnaires were used to assess the effectiveness of cryotherapy. PRO surveys were given out at baseline before paclitaxel therapy, as well as 1-2 weeks, 3-6 months, and 9 months afterward (T0-T4); at baseline, 1-2 weeks, and 6 months after paclitaxel treatment (T0, T1, and T3, respectively); electrophysiological evaluations were carried out
	Population	46 patients; cryotherapy: 23, control group: 23
	Intervention	Cryotherapy was delivered using Elastogel hypothermia gloves and socks (-20 to -10 °C) on both hands and feet 15 minutes before the paclitaxel infusion until 15 minutes post-infusion, for a total of 90 minutes
	Outcome	Safety and efficacy: At two weeks following paclitaxel treatment, there was no discernible difference in the severity of the PNQ between cryotherapy and standard care (sensory: p=0.721; motor: p=1.000), PNQ (sensory: 14.3 vs. 41.2%, p=0.078; motor: 0 vs. 29.4%, p=0.012) and CIPN20 (sensory: β = -3.6, 95% CI: -10.5-3.4, p=0.308; motor: β = -7.3, 95% CI: -14.6-0, p=0.051) results indicated a benefit. Additionally, cryotherapy patients had lower autonomic CIPN20 scores (β = -5.84, 95% CI = -11.15 to -0.524, p=0.031) and higher sympathetic skin response hand amplitudes (β = 0.544, 95% CI: 0.108-0.98, p=0.014), which may have autonomic benefits. Adverse effects: Due to a temporary intolerance to cold, 80.9% of the individuals had their cryotherapy treatment interrupted
	Limitation	High rates of interruption during cryotherapy may have jeopardized the accuracy of the effectiveness findings
Chitkumarn et al., 2022 [23]	Study design	A randomized controlled trial conducted at the gynecological oncology ward of the Department of Obstetrics and Gynecology, Faculty of Medicine, Siriraj Hospital, Mahidol University, Bangkok, Thailand
	Summary	79 Patients receiving 12 weekly doses of paclitaxel (175 mg/m^2^ every 3 weeks) to treat various gynecological cancers were randomly assigned to either the cryotherapy or control group. The in-house Siriraj frozen gloves remained in place for 15 minutes prior to and 15 minutes following the administration of paclitaxel. CIPN incidence and symptom severity were assessed using the FACT/GOG-Ntx score
	Population	79 patients; control arm: 40; study arm: 39
	Intervention	Using velvet fabric bags with two pieces of gel packs inside, Siriraj's frozen gloves were created in-house and left in place from 15 minutes before to 15 minutes after the administration of paclitaxel
	Outcome	Safety and efficacy: The incidences of CIPN were 100% and 48.7%, respectively, in the control and cold-therapy groups. Between the first cycle and the 1-month follow-up following the end of chemotherapy, CIPN was considerably reduced in the intervention group (p=0.001). Adverse effects: Four patients discontinued the cold therapy due to pain, but there were no serious adverse effects due to the therapy
	Limitation	The FACT/GOG-Ntx questionnaire is a subjective report that is less reliable than other assessment methods; a short follow-up period

Effectiveness

Ruddy et al. [20] studied the effectiveness of cryotherapy (bags of crushed ice) in breast cancer patients receiving 12 weeks of paclitaxel by using EORTC QLP-CIP20 sensory score, and the difference between the intervention and control groups was statistically non-significant. However, in the study by Shigmetasu et al. [21], who assessed the effectiveness of cryotherapy for the prevention of paclitaxel-induced neuropathy using the FACT-NTX score, the percentage of patients with a marked decrease in FACT-NTX scores was significantly lower in the cryotherapy group than in the control group. The incidence of National Cancer Institute-Common Terminology Criteria for Adverse Events (NCI-CTCAE) grade ≥2 sensory and motor peripheral neuropathy, and PNQ grade D or higher for sensory peripheral neuropathy, and the decrease in the FACT-Taxane score were also significantly lower in the cryotherapy group compared to the control group. Ng et al. [22] found that cryotherapy was effective at three months post-paclitaxel administration based on lower PNQ and CIPN20. Additionally, patients who underwent cryotherapy had lower CIPN20 autonomic scores and higher sympathetic skin response hand amplitudes, indicating possible autonomic benefits from the procedure. Moreover, Chitkumaran et al. [23] discovered that the intervention group's CIPN considerably decreased during the first cycle and at the one-month follow-up following the discontinuation of chemotherapy (p=0.001).

Safety

None of the studies reported any serious adverse events in patients receiving cryotherapy for the prevention of paclitaxel-induced neuropathy. The main adverse effects were redness, pain, numbness/tingling, and cold intolerance. No severe adverse effects like frostbite, frozen limbs, swelling, skin infection, or scarring were reported in any of the four studies.

Discussion

Summary of Findings

Paclitaxel, a commonly used chemotherapy drug, has demonstrated significant efficacy in treating various types of cancer. However, a notable concern with its use is the development of PIPN, which often leads to dosage reduction and treatment interruptions. Hence, effective therapy is needed to prevent paclitaxel-induced neuropathy. This systematic review aimed to analyze the effectiveness and safety of cryotherapy for the prevention of PIPN. Cryotherapy's effectiveness in preventing CIPN was variable overall, with almost half of the included studies showing a meaningful improvement in at least one outcome.

Ruddy et al. [20], who studied the effectiveness of cryotherapy in 46 breast cancer patients receiving 12 weeks of paclitaxel using EORTC QLP-CIP20 sensory score, found the difference between the study and control arms to be statistically non-significant, with a mean difference of 3.45 (p=0.26). When the cryotherapy arm of the trial was compared to the combined control arm (minocycline and pregabalin) during the 12-week treatment period and at six months following the completion of treatment, they found a p-value of 0.01 based on the Wilcoxon rank-sum test, indicating that there was less neuropathy seen in cryotherapy arm than in the combined control arms. Similarly, in the study by Ng et al. [22], there was no significant difference in PNQ severity between cryotherapy and usual care at two weeks after paclitaxel treatment (sensory: p=0.721; motor: p=1.000). A benefit was observed at three months post-paclitaxel based on PNQ and CIPN20, but it was not sustained in the long term, which further limited the clinical significance of the intervention. Cryotherapy patients did report better overall quality of life at nine months after receiving paclitaxel (β=10.691, 95% CI: 1.5-19.881, p=0.024), but this should not be taken as evidence of a reduction in neuropathic symptoms. Cryotherapy patients did not consistently report higher HRQoL compared to control participants over time. This study's shortcomings may mostly be attributed to the small sample size and the high number of cryotherapy interruptions (80.9%).

A similar study was performed by Shigematsu et al. [21], on 44 breast cancer patients to analyze the effectiveness of cryotherapy. Their results revealed that the percentage of patients with a marked decrease in FACT-NTX scores was significantly lower in the cryotherapy group compared to the control group (41 vs. 73%, p=0.03). Moreover, the incidence of CTCAE grade ≥2 sensory (p=0.001) and motor peripheral neuropathy (p=0.01), and PNQ grade D or higher for sensory peripheral neuropathy (p=0.02), and decrease in the FACT-Taxane score (p=0.02) were also significantly lower in the cryotherapy arm when compared to the control group. Also, they found no serious side effects associated with cryotherapy.

Chitkumarn et al. assessed the efficacy of cold therapy in alleviating paclitaxel-induced peripheral neuropathy. They found that neuropathy was significantly decreased in the intervention group between the first cycle and the one-month follow-up after chemotherapy termination (p=0.001). They also documented no serious adverse effects following the therapy.

The cryotherapy method used predominantly in the studies involved frozen gloves or stocking, except by Ruddy et al. [20], which was the only trial that utilized crushed ice, which was administered 15 minutes before the procedure until 15 minutes after the procedure (total duration: 90 minutes). Prophylactic cryotherapy appears to have few adverse effects in terms of safety. The most frequent negative effects associated with cryotherapy were moderate pain, numbness, tingling, redness, and skin irritation. There were no serious adverse effects detected following cryotherapy. In addition, in the study by Ruddy et al., 14.3% had to have cryotherapy altered due to side effects for at least one cycle [20]. Similar results were found by NG et al., where 80.9% of the individuals experienced a temporary interruption with cryotherapy due to cold intolerance [22].

Based on the above findings, our review shows that cryotherapy reduces the incidence of neuropathy. Cryotherapy also seems to be well tolerated and has few negative side effects. Prophylactic cryotherapy would still be a suitable non-pharmacological method to test for the prevention of CIPN in the absence of a serious adverse effect. To more clearly characterize the function of cryotherapy for the prevention of paclitaxel-induced neuropathy, larger trials that employ consistent cryotherapy administration procedures are required.

Agreement With Contemporary Research

This review's findings are in line with recent discoveries related to PIPN and the prospective application of cryotherapy to its treatment. As noted in this study, it is well-accepted that PIPN is a substantial and dose-limiting adverse effect of paclitaxel treatment [24]. Along with noting the wide range of PIPN prevalence reported in the literature, our exploration of the variances in PIPN prevalence rates across various cancer types and treatment protocols resonates with the complexity of this ailment and the level of research at this time [25].

The review's emphasis on cryotherapy as a possible non-pharmacological method of treating PIPN is consistent with ongoing efforts to develop potent treatments for this serious side effect [26]. Additionally, the description of the likely mechanisms underpinning cryotherapy's effectiveness, including its function in lowering inflammation, repressing axon degeneration, and controlling cytokine release, is consistent with the available knowledge and theories in this field [27].

This review endorses the prevailing agreement regarding the safety and tolerability of cryotherapy, which is typically safe with few side effects like numbness, tingling, and skin irritation [28].

Disagreements and Points of Consideration

This review clarifies particular points of controversy and subject areas that demand more research. Variable study results and the variable effectiveness of cryotherapy for the prevention of PIPN highlight the need for a thorough assessment of its advantages [29]. Furthermore, a persistent research challenge is raised by the discussion surrounding the durability of cryotherapy's benefits, which are shown by transient improvements in some investigations [30].

The differences in sample sizes and interruption rates reported between studies, as shown in this review, may have an effect on how effectively cryotherapy is understood overall [31]. As previously indicated, methodological variation in cryotherapy techniques emphasizes the necessity of standardized protocols for reliable comparisons and insightful conclusions [32].

It is crucial to take into account the intervention's overall effects when interpreting the better quality of life in cryotherapy patients without a clear link to the relief of neuropathic symptoms [33].

Way Forward

This systematic review offers helpful insights that highlight the complexity of researching strategies for PIPN management while also reaffirming existing information. This study adds to the current discussion in the field and highlights the requirement for bigger, carefully planned studies with standardized methods for administering cryotherapy [34]. The dynamic nature of scientific inquiry is reflected in the evolving understanding of the mechanisms behind cryotherapy and its possible long-term repercussions, which opens up new directions for investigation [30]. This review encourages further investigation of successful tactics in this crucial area of cancer care by identifying both points of agreement and avenues for consideration. It also advances knowledge in PIPN management.

Research Implications

The use of paclitaxel, a powerful chemotherapeutic drug, has significantly advanced the treatment of cancer, but it often leads to a negative side effect known as PIPN, which causes pain, numbness, tingling, and loss of sensation in the extremities [20,21]. PIPN prevalence ranges from 30 to 85% in ovarian cancer patients receiving paclitaxel [23] and from 60 to 70% in breast cancer patients [20] depending on the disease type, dose regimens, and patient characteristics. A promising non-pharmacological strategy for controlling PIPN has emerged: cryotherapy [22]. Shigematsu et al. [21] observed considerably lower FACT-NTX scores in the cryotherapy group in comparison to Ruddy et al.'s study [20], which revealed no significant difference in the effectiveness of cryotherapy based on EORTC QLP-CIP20 scores. Based on PNQ and CIPN20 scores, Ng et al. [22] reported benefits of cryotherapy three months after paclitaxel administration. Although occasional discomfort and brief disruptions have been recorded, cryotherapy has typically been found to be safe and effective [20,22]. Additionally highlighting the significance of controlling chemotherapy-induced peripheral neuropathy (CIPN), the American Society of Clinical Oncology offers recommendations for its prevention and management [2].

Strengths and Limitations

We conducted this systematic study by rigorously following the PRISMA guidelines. Only RCTs were considered for this review. The listed articles' bias risk was rigorously assessed using the Cochrane ROB evaluation approach.

However, this study has a few limitations. We only considered articles published in the English language for inclusion. Hence, we may have missed out on significant findings of the studies published in other languages. Secondly, we only incorporated a few RCTs with sufficient sample sizes. As a result, the results occasionally may have been overstated.

## Conclusions

The significance of PIPN as a painful and dose-limiting side effect of paclitaxel treatment is highlighted in this systematic review. Devising efficient therapies is necessitated by the variability in PIPN prevalence among cancer types, dosage regimens, and patient characteristics. Studies have shown inconsistent but encouraging results with regard to cryotherapy, which has emerged as a promising non-pharmacological treatment. While some trials did not reveal a substantial reduction in PIPN symptoms using specific outcome measures, some did, underlining the need for more research. Patient safety is a serious concern, and cryotherapy has a relatively good safety record, despite occasional brief discomfort and disruptions. Cryotherapy may have advantages beyond symptom relief, as suggested by reports of enhanced quality of life in some patients. Nevertheless, reaching firm conclusions has been difficult due to methodological inconsistencies, sample size differences, and disparities in study results.

To determine the true effectiveness of this strategy, larger, more carefully planned trials using standardized cryotherapy methods are required going forward. Future studies are critical given the evolving understanding of the mechanisms behind cryotherapy and its potential long-term impacts. We believe this review advances our knowledge of this crucial part of cancer care by examining both points of agreement and disagreement in the ongoing search for appropriate PIPN management regimens. However, the role of cryotherapy in enhancing the lives of patients receiving paclitaxel treatment will ultimately be determined by the findings of future research.
